# Quantifying the effect of investors’ attention on stock market

**DOI:** 10.1371/journal.pone.0176836

**Published:** 2017-05-23

**Authors:** Zhen-Hua Yang, Jian-Guo Liu, Chang-Rui Yu, Jing-Ti Han

**Affiliations:** 1 School of Information Management Engineering, Shanghai University of Finance and Economics, Shanghai 200433, PR China; 2 Data Science and Cloud Service Research Centre, Shanghai University of Finance and Economics, Shanghai 200433, PR China; 3 Business School, Huzhou University, Huzhou 313000, PR China; 4 Department of Physics, Fribourg University, CH-1700 Fribourg, Switzerland; East China University of Science and Technology, CHINA

## Abstract

The investors’ attention has been extensively used to predict the stock market. Different from existing proxies of the investors’ attention, such as the *Google trends*, *Baidu index* (*BI*), we argue the collective attention from the stock trading platforms could reflect the investors’ attention more closely. By calculated *the increments of* *the attention volume* *for each stock* (*IAVS*) from the stock trading platforms, we investigate the effect of investors’ attention measured by the *IAVS* on the movement of the stock market. The experimental results for *Chinese Securities Index 100* (CSI100) show that the *BI* is significantly correlated with the returns of CSI100 at 1% significance level only in 2014. However, it should be emphasized that the correlation of the new proposed measure, namely *IAVS*, is significantly at 1% significance level in 2014 and 2015. It shows that the effect of the measure *IAVS* on the movement of the stock market is more stable and significant than *BI*. This study yields important invest implications and better understanding of collective investors’ attention.

## Introduction

The investors’ attention plays an important role in predicting the movement of the stock market, which has attracted much attention recently [1–4]. The attention is not only a scarce cognitive resource [5] but also the hard currency of cyberspace [6]. In fact, it is hard for most investors, especially retail investors, to access the market information timely and accurately. Therefore, most investors would like to pay more attention to the attracted information to adjust their investment behavior, leading to the movement of the stock market [7, 8].

The proxies of investors’ attention for predicting the movement of the stock market could be roughly classified into two categories. The indirect proxies of investors’ attention mainly include extreme return [9], trading volume [10–14], turnover [15, 16], etc. [17–20], which have been extensively analyzed by professional investors for many years. On the other hand, direct proxies of investors’ attention, such as *search volume index* (*SVI*) [1, 21–34], social network (Twitter feeds, blogs, forum, Wikipedia etc.) [35–42], news [43–51], etc. have been introduced to predict the movement of the stock market.

In particular, the massive data sources resulting from human interaction with the Internet have offered a new perspective on the behavior of market participants besides investors in the stock market. For example, the *SVI* has been used to predict the movement of the stock market [1, 21–26,30–34]. By introducing the search volume in Google of a sample of Russell 3000 stocks, Da *et al*. [1] found that the increase in the *SVI* could successfully predict higher stock prices in a short term and eventual price reversal. Based on the *Google trends* or *Baidu index*, the similar results of French, Japanese and Chinese stock markets have been found [32–34]. Preis *et al*. [21] found that the search volume of the *Google trends* for financial related words could be used to predict the stock market volatility. Besides the *SVI*, the sentiment detected from the social network users also could affect the movement of the stock market [35–40]. By the inclusion of specific public mood dimensions, Bollena *et al*. [35] found that the prediction accuracy of the Dow Jones Industrial Average can be significantly improved. These works help us to predict the movement of the stock market in the new perspectives. However, in the data generated from search engine, different users always input the same keywords with different motivations. Even for the exact same motivation, two users always input different keywords. For the sentiment of the social networks, the sentiment need to be calculated based on the ontology or domain dictionaries, which could not updated dynamically in terms of the stock market, leading to biased prediction. In this paper, we argue that the data generated from the stock trading platforms can reflect the investors’ attention more accurately and effectively.

**Fig 1:**
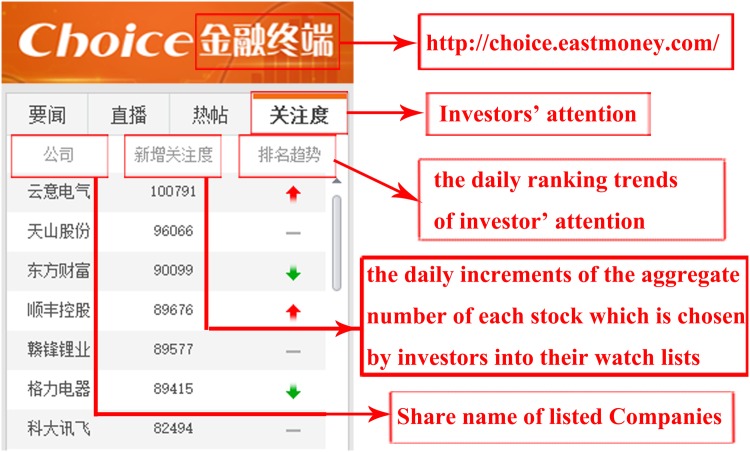
Illustration of *investors’ attention of the stock market* on 2017-03-04. This figure gives an illustration of investors’ attention of stock market column on 2017-03-04. This figure is intercepted from the stock trading platform named Choice, available online at http://choice.eastmoney.com/. As shown, it mentions the top stocks chosen by investors into their watch lists on this stock trading platform on 2017-03-04, the increments and the ranking trend of investors’ attention. In fact, we can get the increments and the ranking trend of investors’ attention of each stock in every trading day in mainland China by collaborating with the eastmoney.com. Please note that the data publishes at 0 o’clock at the same trading day on the stock trading platform.

Inspired by the idea, by taking into account the increments of the attention volume for each stock on the stock trading platforms, we present a new measure *IAVS* to quantify the investors’ attention for predicting the movement of the stock market. The measure *IAVS* introduced in this paper refers to the daily increments of the aggregate number of each stock which is chosen by investors into their watch lists in a stock trading platform. Fig 1 presents an illustration of *investors’ attention of stock market* on 2017-03-04. Firstly, we quantify the effect of the investors’ attention on the returns of CSI100 using the *BI* and the *IAVS* to measure investors’ attention respectively. The results show that the *BI* is significantly correlated with the returns of CSI100 at 1% significance level only in 2014. However, the correlation of the new proposed measure, namely *IAVS*, is significantly at 1% significance level in 2014 and 2015. Secondly, we investigate the relationship between the investors’ attention and the trading activities in the stock market. The results indicate that the measure *IAVS* is correlated with the turnover of CSI100 at 5% significance level in 2014. In addition, we test the robustness with other indices in the stock market, including CSI500 and CSI-ALL, the results show that the *IAVS* is correlated with the returns of CSI500 and CSI-ALL at 1% significance level in 2014 and the *IAVS* is correlated with the returns of CSI-ALL at 5% significance level in 2015. Different from existing proxy *BI* of the investors’ attention, the effect of the measure *IAVS* on the stock market is more stable and significant.

## Theoretical background and hypotheses development

**Fig 2:**
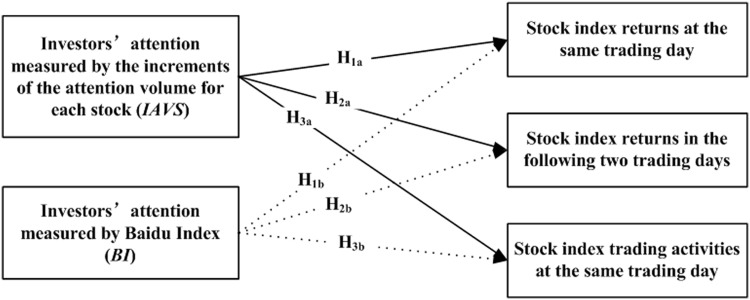
Illustration of the hypotheses structures presented in this paper.

We study the effect of investors’ attention on the stock market with different measures at three levels in this work. Firstly, we investigate the effect of investors’ attention on the returns of CSI100, CSI500, CSI-ALL at the same trading day. Secondly, we uncover the impact of investors’ attention on the returns of CSI100, CSI500, CSI-ALL in the following two trading days. Finally, we testify the relationship between the investors’ attention and the trading activities in the stock market at the same trading day. Fig 2 gives the illustration of the hypotheses structures presented in this paper.

### 2.1 The effect of investors’ attention on stock index returns at the same trading day

How does an increase in retail investors’ attention affect the stock index returns? Barber and Odean [9] argued that individual investors are net buyers of attention-grabbing stocks and thus an increase in individual investors’ attention results in temporary positive price pressure. The reason behind their argument goes as follows. When individual investors are buying, they have to choose from a large set of available alternatives. However, when they are selling, they can only sell what they own. Within the framework of Barber and Odean [9], a positive investors’ attention should predict higher stock prices in the short term. This assumption has been verified in the study of Da *et al*. [1] with the American stock market. Thus, we propose the following hypotheses:

**H**_1*a*_: Increased investors’ attention measured by the *IAVS* will lead to higher returns of corresponding stock index at the same trading day.
**H**_1*b*_: Increased investors’ attention measured by the *BI* will lead to higher returns of corresponding stock index at the same trading day.

### 2.2 The impact of investors’ attention on stock index returns in the following trading days

After the retail investors have made a purchase, professional investors and institutional investors holding high-attention stocks may be aware of the price premium that is included in their price, and they may sell the stock for a premium. When the retail investors’ attention is distracted by other stocks, the price of high-attention stocks may reverse. The results of relative studies [32–34] confirmed that while an increase in investors’ attention brings higher stock prices, the price may reverse in a short term with the samples of French, Japanese and Chinese stock markets. Thus, we propose the following hypotheses:

**H**_2*a*_: Increased investors’ attention measured by the *IAVS* will impact on the returns of corresponding stock index significantly in the following trading days.
**H**_2*b*_: Increased investors’ attention measured by the *BI* will impact on the returns of corresponding stock index significantly in the following trading days.

### 2.3 The relationship between the investors’ attention and trading activities in stock market

Over recent years, researchers have found that psychological factors including attention appear to have significant influences on the trading decisions of investors within the financial markets, and thus, on both returns and trading activities [52–54]. Trading volume and turnover are two important indicators of the trading activities to describe the liquidity of the stock market. Once an investor pay attention to some stocks, he or she would probably put these stocks into their watch list termed, which will inevitably affect their expectations and behavior of investment. And the final performance is there will be correlation between the investors’ attention and trading activities in the stock market. Yu and Zhang [49] argued that the stocks with active performance of trading activities will cause more investors’ attention, thereby strengthen the correlation between the investors’ attention and trading activities [33]. Li argued that the stocks with high trading volume bring more attention in news reports. Thus, we propose the following hypotheses:

**H**_3*a*_: The investors’ attention measured by the *IAVS* has associated significantly with the trading activities of corresponding stock index.
**H**_3*b*_: The investors’ attention measured by the *BI* has associated significantly with the trading activities of corresponding stock index.

## Data collection and measures for variables

### 3.1 Data collection and analysis

In this paper, we introduce three databases, including search volume index of *Baidu Index* (http://index.baidu.com/) termed *BI* as benchmark, investors’ attention on stock trading platform (http://choice.eastmoney.com/) termed the *IAVS*, and stock transaction data from the stock trading platform (http://choice.eastmoney.com/). The Baidu search engine is the largest Chinese search engine in the world at present, and eastmoney.com is one of the largest and most influential financial trading platform in mainland China with approximately 1.6 million active users daily and more than one hundred million users so far. The *BI* is generated on *Baidu Index* for the keywords of the names and codes of each stock; the measure *IAVS* is generated from eastmoney.com for the increments of attention volume of each stock. To match the *BI* and the measure *IAVS* to the respective time series of the stock market we only consider trading days of the stock markets in this paper.

**Fig 3:**
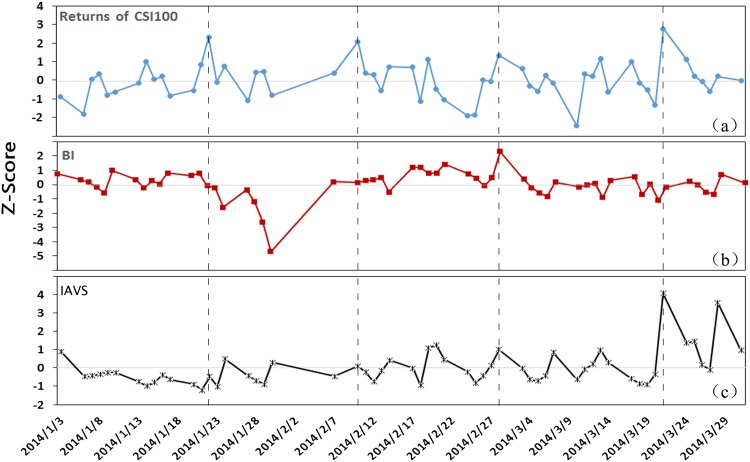
The illustration of the correlation between the CSI100 returns and the investors’ attention measured by the *BI* and *IAVS* for the day. The subplots (a)-(c) show the time series of Z-score of the returns of CSI100, the *BI* and the measure *IAVS* respectively, from which one can find that the measure *IAVS* correlates with the returns of CSI100 more closely. The Z-score is calculated in the way *z* = (*x* − *μ*)/*σ*, where *x*, *μ* and *σ* denote the original, mean value and standard deviation. We only consider trading days of the stock market, so there is no data in weekends and holidays.

This work focuses on the three major Chinese stock indices, including CSI100, CSI500, and CSI-ALL, in the Shanghai Stock Exchange(SSE) and Shenzhen Stock Exchange(SZSE) in mainland China from January 3, 2014 to December 31, 2015, when is the only period that we can get the data for CSI100 from Baidu Index due to the service limitations of Baidu Index. CSI100 consists of the largest 100 stocks in mainland China; CSI500 reflects the overall situation of small cap companies in Chinese stock markets; CSI-ALL consists of all securities in mainland China. We use CSI100 to quantify the effect of the investors’ attention on the movement of the stock market and compare the results between the *IAVS* and *BI*. Then we use CSI500 and CSI-ALL to test the robustness of the results based on the *IAVS* as a measure of the investors’ attention. Fig 3 gives the illustration of the correlation between the CSI100 returns and the investors’ attention respectively measured by the *BI* and the *IAVS*. We also provide explanation about the measures of this work in more detail in the rest of this section (The minimal data set is included in S1 and S2 Data).

#### Stationarity tests

To cover various combinations of relationships, we initially study standard transformations of the original series, i.e. the first differences. For each of the series, we test their stationarity using the Augmented Dickey-Fuller test (ADF) [55] and the KPSS [56] test. As both tests have opposite null and alternative hypotheses, they form an ideal pair for the stationarity vs. unit-root testing [42]. For the *BI_i,t_*, we find the original series to be non-stationary and to contain the unit-root. Correspondingly, its first differences are stationary. The same results are found for the *Volume_i,t_*, *Turnover_i,t_*, we find the unit-root only for the first differences transformation of the series mentioned above. For this reason and also for more convenient interpretation, we opt for the first differences series. And the original series *IAVS_i,t_* is not always stationary in different indices, so we opt for the first differences of the series *IAVS_i,t_* (see detailed discussion in S1 Text and S1 Table).

#### Serial correlation tests

Turning now to the analysis of the serial correlation. At the beginning, the autocorrelation coefficient, partial autocorrelation coefficient and Q statistic of the residual series are calculated. If the corresponding p-value of the test is less than 0.05, the null of no serial correlation is rejected and, therefore, it can be concluded that there might be serial correlation in the returns [57, 58]. If the residual series of the regression equation has serial correlation, it is necessary to modify the autocorrelation of the residuals in a correct way. There is no serial correlation among all the modified regression equations, the estimated results of which are valid (see detailed discussion in S2 Text, S1, S2, S3 and S4 Figs)

### 3.2 Independent variables

***BI**_i,t_*. We construct a time series of daily aggregate search volume of each stock listed in the index *i* on *Baidu Index* as the following way:

$$\begin{array}{c} BI_{i,t}=\sum_{j=1}^{n}\left(BIname_{j,i,t}+BIcode_{j,i,t}\right), \end{array}$$ (1)

where *BIname*_*j*,*i*,*t*_ and *BIcode*_*j*,*i*,*t*_ are the aggregate search volume through its name or code of stock *j* in the list of stock index *i* on day *t* via *Baidu Index*, *n* is the total number of the stocks listed in stock index *i*. We use *D_BI_i,t_* indicate the first difference of the series *BI_i,t_*.

***IAVS**_i,t_*. We construct a time series of daily attention increments of stocks in the index *i* on the stock trading platform as the following way:

$$\begin{array}{c} IAVS_{i,t}=\sum_{j=1}^{n}IAVS_{j,i,t}, \end{array}$$ (2)

where *IAVS_j,i,t_* is the daily attention increments of stock *j* which is in the list of stock index *i* on day *t* via the stock trading platform. That is to say if one investor add stock *j* into his watch list on day *t*, the attention increments of stock *j* will be added by 1 on day *t* on the stock trading platform. And *n* is the total number of stocks listed in index *i*. Also, we use *D_IAVS_i,t_* indicate the first difference of *IAVS_i,t_*.

### 3.3 Dependent variables

***Return**_i,t_*. We construct a time series for daily returns of the stock indices without deduction of charges as the following way:

$$\begin{array}{c} Return_{i,t}=\frac{\left(Closing\;price_{i,t}-Closing\;price_{i,t-1}\right)}{Closing\;price_{i,t-1}}\times 100\%, \end{array}$$ (3)

where *Closing price*_*i*,*t*_ is the closing price of index *i* on day *t*, and day *t-1* is the trading day before day *t*. Also, we use *D_Return_i,t_* indicate the first difference of the series *Return_i,t_*.

***Volume**_i,t_*. We use this variable to describe the trading volume of index *i* on day *t*. Trading volume measures the total number of shares or indices transacted for a specified security during a specified time period. It includes the total number of indices transacted between a buyer and seller during a transaction. When indices are more actively traded, their trade volume may high, and when indices are less actively traded, their trade volume may low. Similarly, we use *D_Volume_i,t_* indicate the first difference of the series *Volume_i,t_*.

***Turnover**_i,t_*. We use this variable to describe the turnover rate of index *i* on day *t*. The turnover rate is the percentage of a mutual fund or other investment’s holdings that have been replaced in a given period. Generally speaking, high turnover rate of index means that the index has strong liquidity. Similarly, we use *D_Turnover_i,t_* indicate the first difference of the series *Turnover_i,t_*.

Table 1 gives the variable description used in this paper.

**Table 1:** Description of variables.

Variable	Description
***BI***_*i*,*t*_	*The aggregate search volume number of all stocks listed in stock index i* *on day t released at 24 o’clock at the same trading day on Baidu Index*
***IAVS***_*i*,*t*_	*The Increments of the Attention Volume of all Stocks in stock index i* *in all investors’ watchlist termed on day t released at 0 o’clock at the* *same trading day on stock trading platforms*
***Return***_*i*,*t*_	*The returns of stock index i on day t without deduction of charges*
***Volume***_*i*,*t*_	*The trading volume of stock index i on day t*
***Turnover***_*i*,*t*_	*The turnover rate of stock index i on day t*

## Results

By using Single-Variable Regression Analysis in time series, we test the relationship between the investors’ attention and the movement of the stock market. The investors’ attention was measured by two different proxies, the *BI* and the *IAVS*, to compare with each other. First, we estimate the Model I to testify the effect of the *BI* on the returns of stock index.

$$\begin{array}{c} Return_{i,s}=\beta_{1}+\beta_{2}D\_BI_{i,t}+\varepsilon_{i,s}. \end{array}$$ (I)

Also, we estimate the Model II to testify the effect of the *IAVS* on the returns of stock index.

$$\begin{array}{c} Return_{i,s}=\beta_{3}+\beta_{4}D\_IAVS_{i,t}+\varepsilon_{i,s}, \end{array}$$ (II)

where *s* vary from *t* to *t+1, t+2*, that is, we use the variables of investors’ attention on day *t* to predict the returns of the very trading day *t* and the following two trading days day *t+1*, day *t+2*.

Then, we estimate the Model III to testify the relationship between the investors’ attention measured by the *BI* and the *Trading_Activities*. The *Trading_Activities_i,t_* includes the *D_Turnover_i,t_* and *D_Volume_i,t_*.

$$\begin{array}{c} Trading\_Activities_{i,t}=\beta_{5}+\beta_{6}D\_BI_{i,t}+\varepsilon_{i,t}. \end{array}$$ (III)

Also, we estimate the Model IV to testify the relationship between the investors’ attention measured by the *IAVS* and the *Trading_Activities*. Similarly, the *Trading_Activities_i,t_* includes the *D_Turnover_i,t_* and *D_Volume_i,t_*.

$$\begin{array}{c} Trading\_Activities_{i,t}=\beta_{7}+\beta_{8}D\_IAVS_{i,t}+\varepsilon_{i,t}. \end{array}$$ (IV)

### 4.1 Results for CSI100

The sample in this section includes 73 stocks with no missing data in CSI100 with 485 daily observations from January 3, 2014 to December 31, 2015. Particularly, we test the impact of the investors’ attention for day *t* on the the stock index returns for *day t, day t+1, day t+2* in order to find whether the investors’ attention has prediction effect on the movement of the stock market in the short term.

Table 2 shows the results of the regression modeling with CSI100. Firstly, the results show that the coefficients of *Return_i,t_* are positive and significant at 1% significance level for *D_IAVS_i,t_* and *D_BI_i,t_* in 2014, supporting the hypotheses **H**_1*a*_ and **H**_1*b*_, which indicate that the more investors’ attention of the stocks, the higher returns of the corresponding stock index at the same trading day. Specifically, the *D_IAVS_i,t_* is released at 0 o’clock on day *t* which is before the opening time of the stock market on day *t*. However, the *D_BI_i,t_* is generated at 24 o’clock on day *t* which is after the closing time of the stock market on day *t*. So the *IAVS* may has prediction effect on the movement of stock market at the same trading day.

**Table 2:** Results of the regression modeling with CSI100.

Variables	*D*_*BI_i,t_* (2014)	*D*_*IAVS_i,t_* (2014)	*D*_*BI_i,t_* (2015)	*D*_*IAVS_i,t_* (2015)
***Return***_*i*,*t*_	0.201** (0.009)	0.419** (0.000)	-0.147 (0.138)	0.261** (0.000)
***Return***_*i*,*t*+1_	0.533** (0.000)	-0.360** (0.000)	-0.058 (0.597)	-0.141* (0.011)
***Return***_*i*,*t*+2_	0.181 (0.087)	0.193* (0.045)	-0.006 (0.783)	0.031 (0.639)
***D_Turnover***_*i*,*t*_	-0.000 (0.983)	0.234* (0.026)	-0.032 (0.289)	0.181 (0.087)

Note:

**p < 0.01,

*p < 0.05

Secondly, the coefficients of *Return*_*i,t*+1_ are significant at 1% significance level for *D_IAVS_i,t_* and *D_BI_i,t_* in 2014, supporting the hypotheses **H**_2*a*_ and **H**_2*b*_. Specifically, only the coefficients of *Return*_*i,t*+2_ are significant at 5% significance level for *D_IAVS_i,t_* in 2014, supporting the hypotheses **H**_2*a*_, which suggest that increased investors’ attention measured by the *IAVS* will impact on the returns of corresponding stock index significantly in the following two trading days.

Thirdly, the coefficients of *D_Turnover*_*i,t*_ are significant at 5% significance level for *D_IAVS_i,t_* in 2014, supporting the hypotheses **H**_3*a*_. Different from the *BI*, the measure *IAVS* has associated significantly with the *Turnover* of corresponding stock index at the same trading day. According to the results, we find the effects of investors’ attention with different proxies on the movement of the stock market in the following trading days are different. A reason may be the different proxies are generated by different users, the *IAVS* is generated on stock trading platform where the users are more professional in investment than the users on search engine web.

It should be noticed the coefficients of *Return_i,t_* are significant at 1% significance level for *D_IAVS_i,t_* in 2015, providing support for **H**_1*a*_. And the coefficients of *Return*_*i,t*+1_ are significant at 5% significance level for *D_IAVS_i,t_* in 2015, providing support for **H**_2*a*_. However, the results from the *BI* are not significant in 2015. The possible reason is that the stock market is changed a lot between 2014 and 2015, and it is in the bullish market in 2014 and volatile market in 2015 in mainland China. From the empirical results, we find the measure *IAVS* contributes to the forecasting for the movement of CSI100 persistently no matter how the market changes. Generally speaking, with the sample in this work, the results from the *IAVS* outperforms the results of the *BI*.

### 4.2 Results for CSI500 and CSI-ALL

We explore the effect of investors’ attention on the movement of the stock market by using different stock indices, including CSI500 and CSI-ALL, to provide a cross-category robustness check. The data sets contain 485 daily observations from January 3, 2014 to December 31, 2015.

### Empirical analysis based on CSI500

Table 3 shows the results of the Regression Modeling with CSI500. The coefficients between the independent variables *D_IAVS_i,t_* and the variables of the stock index, including *Return_i,t_*, *Return*_*i,t*+1_, *Return*_*i,t*+2_, *D_Volume*_*i,t*_ and *D_Turnover*_*i,t*_ for CSI500 are all significant at 5% significance level for *D_IAVS_i,t_* in 2014, supporting the hypotheses **H**_1*a*_, **H**_2*a*_, **H**_3*a*_. The results indicate that the more investors’ attention on the stocks of CSI500, the higher returns in the first three trading days and active trading activities at the same trading day of CSI500 in 2014. Although the coefficients between the *D_IAVS_i,t_* and the movement of the stock index in 2015 are not significant, the direction is the same as its in 2014.

**Table 3:** Results of the regression modeling with CSI500.

Variables	*D*_*IAVS_i,t_*(2014)	*D*_*IAVS_i,t_*(2015)
***Return***_*i*,*t*_	0.118** (0.009)	0.164 (0.155)
***Return***_*i*,*t*+1_	0.284* (0.022)	0.104 (0.370)
***Return***_*i*,*t*+2_	0.214* (0.013)	0.102 (0.482)
***D_Volume***_*i*,*t*_	0.878** (0.000)	0.076 (0.332)
***D_Turnover***_*i*,*t*_	0.146** (0.000)	0.175 (0.346)

Note:

**p < 0.01,

*p < 0.05

Comparing the results of CSI500 with CSI100, we find that the measure *IAVS* is more efficient to the stock index with large firms than small firms in volatile market. This results are different from the argument posited by GM Mian and Sankaraguruswamy(2008) that the influence of sentiment on the stock price response is especially pronounced for small stocks [51]. Both results tell us that the investors’ attention is not the same as the sentiment of investors, and the detail research need to be explored in future studies.

### Empirical analysis based on CSI-ALL

Table 4 presents the results of the Regression Modeling with CSI-ALL. The coefficients between the independent variables *D_IAVS_i,t_* and the variables of the stock index, including *Return_i,t_*, *Return*_*i,t*+1_, *D_Volume*_*i,t*_ and *D_Turnover*_*i,t*_ for CSI-ALL are all significant at 1% significance level for *D_IAVS_i,t_* in 2014, providing support for **H**_1*a*_, **H**_2*a*_, **H**_3*a*_. The results indicate that the more investors’ attention on the stocks of CSI-ALL, the higher returns in the first three trading days and active trading activities at the same trading day of CSI-ALL in 2014.

**Table 4:** Results of the regression modeling with CSI-ALL.

Variables	*D*_*IAVS_i,t_*(2014)	*D*_*IAVS_i,t_*(2015)
***Return***_*i*,*t*_	0.109** (0.000)	0.090* (0.019)
***Return***_*i*,*t*+1_	0.115** (0.000)	0.006 (0.783)
***Return***_*i*,*t*+2_	0.107** (0.000)	-0.015 (0.742)
***D_Volume***_*i*,*t*_	0.641** (0.000)	0.256* (0.010)
***D_Turnover***_*i*,*t*_	0.175** (0.000)	0.089* (0.020)

Note:

**p < 0.01,

*p < 0.05

In addition, the coefficients between the independent variables *D_IAVS_i,t_* and the variables of the stock index, including *Return_i,t_*, *D_Volume*_*i,t*_ and *D_Turnover*_*i,t*_ for CSI-ALL are significant at 5% significance level for *D_IAVS_i,t_* in 2015, supporting the hypotheses **H**_1*a*_, **H**_2*a*_, **H**_3*a*_. The results indicate that the more investors’ attention on the stocks of CSI-ALL, the higher returns and active trading activities at the same trading day of CSI-ALL in 2015. Comparing the results between 2014 and 2015, we find the *IAVS* is efficient to the movement of CSI-ALL, although it has experienced a volatile period in Chinese stock market in 2015.

Among the results of CSI-ALL, CSI100 and CSI500, we could find the consistent results in most cases as follows: (1) the effect of investors’ attention measured by the *IAVS* on the returns of the stock index at the same trading day are positive and significant; (2) the relationship between the investors’ attention measured by the *IAVS* and the trading activities of the corresponding stock index at the same trading day are positive and significant; (3) the impact of investors’ attention measured by the *IAVS* on the movement of the stock index with small firms are the same as the stock index with all firms.

## Conclusion and discussions

To accurately predict the movement of the stock market, different with the traditional measures of investors’ attention, including the *Google trend* and *Baidu Index*, we empirically investigate the effect of the investors’ attention, namely *IAVS* generated by investors on the stock trading platforms on the movement of the stock market. The measure *IAVS* is updated rapidly, providing first-hand information to reflect the investors’ attention ahead of other sources. Thus, the attention volume of each stock provides us with a good context to study how to quantify the investors’ attention effectively and what are the influences of investors’ attention on the movement of the stock market.

In this paper, we propose the hypotheses that the investors’ attention will impact on the returns and the trading activities of corresponding stock index in a short time. By using Single-Variable Regression Analysis in time series, we test the relationship between the investors’ attention, measured by two different proxies the *BI* and *IAVS*, and the movement of the stock market. The samples includes CSI100, CSI500 and CSI-ALL with 485 daily observations from 2014 to 2015. The findings in this work show that the results of the measure *IAVS* generated from the stock trading platform is more significant and stable than the *BI* generated from the search engine. The experimental results mainly show that compared with the *BI*, the measure *IAVS* is significant related to the returns of the corresponding stock indices CSI100 and CSI-ALL at the same trading days in 2015; the measure *IAVS* is significant related to the returns of the corresponding stock index in the first three trading days in the bullish market in 2014; the measure *IAVS* has associated significantly with the trading activities of corresponding stock index at the same trading day. From the results, we also get the findings that the effect of the measure *IAVS* on the movement of the stock market may differ depending on the firm’s size, which show that the measure *IAVS* is more significant to the movement of stock index containing large firms; and the effect of the measure *IAVS* on the movement of the stock market is also influenced by the market environment, which show that the results of the bullish market in 2014 are more stable than those of the volatile market in 2015 in mainland China.

From a theoretical perspective, our study enriches extant research by focusing on the relationship between the investors’ attention and the movement of the stock market. Specifically, we developed the measure *IAVS* to quantify the investors’ attention, which also can be readily applied to predict the financial risks or understand the collective investors’ behavior. From a practical perspective, our findings may help retail investors better predict the stock index trends, based upon which they can make better investment decisions. Stock price prediction is an important and challenging problem for studying financial markets, which has been investigated in different ways. Recently, Lin et al. [59] found that the friendship networks and information asymmetry play an important role for the P2P lending system. Therefore, how to construct the trader social network [60] in terms of their trading behavior, and how to construct the stock network in term of the movement of stock pricing [61–63], as well as how to measure the effect of the network structure on the prediction performance are open questions, which would be investigated in our future research direction. Besides the social network structure, for the online traders, the collective holding behaviors [64, 65] and social influence [66] would affect their trading decision. More importantly, to identify the mechanism of the online trading system, predict the movement of stock market, and build the recommendation system [67], we need to investigate the above questions from the viewpoint of complex systems, which could bring new insights and knowledge for deeply understanding the evolution mechanism of the stock market.

## Supporting information

S1 Text The stationarity tests. (PDF)

S2 Text The serial correlation tests. (PDF)

S1 Table Stationarity and unit-root tests. (PDF)

S1 Fig Serial correlation tests between the D_BI_*i*,*t*_ and Return_*i*,*t*_. (JPG)

S2 Fig Serial correlation tests between the D_IVAS_*i*,*t*_ and Return_*i*,*t*_. (JPG)

S3 Fig Serial correlation tests between the D_BI_*i*,*t*_ and Turnover_*i*,*t*_. (JPG)

S4 Fig Serial correlation tests between the D_IVAS_*i*,*t*_ and Turnover_*i*,*t*_. (JPG)

S1 Data The minimal data set for CSI100 in 2014. (XLSX)

S2 Data The minimal data set for CSI100 in 2015. (XLSX)
