# Supplementary material for: Quantifying the effect of investors’ attention on stock market
Source: PLoS One. 2017 May 23;12(5):e0176836. doi: 10.1371/journal.pone.0176836 (PMC5441604; doi:10.1371/journal.pone.0176836)
Supplement: S1 Text — (PDF) [file pone.0176836.s001.pdf]

SUPPORTING INFORMATION S1 TEXT

Zhen-Hua Yang[1,3], Jian-Guo Liu[2,4*], Chang-Rui Yu[1*], Jing-Ti Han[2]

1 School of Information Management Engineering, Shanghai University of Finance and Economics, Shanghai 200433, PR China,
2 Data Science and Cloud Service Research Centre, Shanghai University of Finance and Economics, Shanghai 200433, PR China,
3 Business School, Huzhou University, Huzhou 313000, PR China,
4 Department of Physics, Fribourg University, CH-1700 Fribourg, Switzerland

* E-mail: liujg004@ustc.edu.cn

The Stationarity Tests

In this paper, we introduce the stationarity test using the Augmented Dickey-Fuller test (ADF) [1] and the KPSS test [2, 3]. As both tests have opposite null and alternative hypotheses, they form an ideal pair for the stationarity vs. unit-root testing. ADF has a null hypothesis of a unit root against the alternative of no unit root whereas KPSS has a null of stationarity against an alternative of a unit root. Using the pair of tests, we are able to identify whether the tested series is stationary or not.

To cover various combinations of relationships, we initially study standard transformations of the original series, i.e. the first differences. For each of the series, we test their stationarity using the ADF and the KPSS test. For the $BI_{i,t}$, we find the original series to be non-stationary and to contain the unit-root. Correspondingly, its first differences are stationary. The same results are found for the $Volume_{i,t}$, $Turnover_{i,t}$, we find the unit-root only for the first differences transformation of the series mentioned above. For this reason and more convenient interpretation, we opt for the first differences series. And the original series $IAVS_{i,t}$ is not always stationary in different indices, so we opt for the first differences of the series $IAVS_{i,t}$. We summarize all the results of stationarity tests in Table S1.

References

1. Dickey D A, Fuller W A. Distribution of the estimators for autoregressive time series with a unit root. Journal of the American Statistical Association. 1979; 74(366a): 427-431. doi:http://www.jstor.org/stable/2286348

2. Kwiatkowski D, Phillips P C B, Schmidt P, et al. Testing the null hypothesis of stationarity against the alternative of a unit root: How sure are we that economic time series have a unit root? Journal of Econometrics. 1992; 54(1-3): 159-178. doi:10.1016/0304-4076(92)90104-Y

3. Kristoufek L. BitCoin meets Google trends and Wikipedia: Quantifying the relationship between phenomena of the Internet era. Scientific Reports. 2013; 3(3): 3415. doi:10.1038/srep03415

Table S1. Stationarity and Unit-root Tests.

Series		KPSS	p-value	ADF	p-value
$\boldsymbol{BI}_{i,t}$	CSI100	0.276	<0.01	−2.133	>0.1
$\boldsymbol{D\_BI}_{i,t}$	CSI100	0.044	>0.1	−27.839	<0.01
$\boldsymbol{IAVS}_{i,t}$	CSI100	0.378	<0.01	−3.787	<0.01
	CSI500	0.155	>0.1	−9.468	<0.01
	CSI-ALL	0.242	>0.1	−2.611	0.091
$\boldsymbol{D\_IAVS}_{i,t}$	CSI100	0.036	>0.1	−13.208	<0.01
	CSI500	0.022	>0.1	−16.025	<0.01
	CSI-ALL	0.020	>0.1	−13.462	<0.01
$\boldsymbol{Return}_{i,t}$	CSI100	0.094	0.056	−17.416	<0.01
	CSI500	0.073	>0.1	−18.523	<0.01
	CSI-ALL	0.142	>0.1	−16.342	<0.01
$\boldsymbol{D\_Return}_{i,t}$	CSI100	0.078	>0.1	−15.581	<0.01
	CSI500	0.041	>0.1	−15.469	<0.01
	CSI-ALL	0.021	>0.1	−15.145	<0.01
$\boldsymbol{Volume}_{i,t}$	CSI100	0.376	<0.01	−2.424	>0.1
	CSI500	0.318	<0.01	−2.923	0.043
	CSI-ALL	0.360	<0.01	−2.592	0.095
$\boldsymbol{D\_Volume}_{i,t}$	CSI100	0.052	>0.1	−17.113	<0.01
	CSI500	0.089	>0.1	−21.154	<0.01
	CSI-ALL	0.054	>0.1	−20.024	<0.01
$\boldsymbol{Turnover}_{i,t}$	CSI100	0.377	<0.01	−2.485	>0.1
	CSI500	0.366	<0.01	−3.138	0.025
	CSI-ALL	0.375	<0.01	−2.637	0.086
$\boldsymbol{D\_Turnover}_{i,t}$	CSI100	0.058	>0.1	−16.745	<0.01
	CSI500	0.079	>0.1	−20.923	<0.01
	CSI-ALL	0.052	>0.1	−19.958	<0.01
