# Supplementary material for: Quantifying the effect of investors’ attention on stock market
Source: PLoS One. 2017 May 23;12(5):e0176836. doi: 10.1371/journal.pone.0176836 (PMC5441604; doi:10.1371/journal.pone.0176836)
Supplement: S2 Text — (PDF) [file pone.0176836.s002.pdf]

SUPPORTING INFORMATION S2 TEXT

Zhen-Hua Yang[1,3], Jian-Guo Liu[2,4*], Chang-Rui Yu[1*], Jing-Ti Han[2]

1 School of Information Management Engineering, Shanghai University of Finance and Economics, Shanghai 200433, PR China,
2 Data Science and Cloud Service Research Centre, Shanghai University of Finance and Economics, Shanghai 200433, PR China,
3 Business School, Huzhou University, Huzhou 313000, PR China,
4 Department of Physics, Fribourg University, CH-1700 Fribourg, Switzerland

* E-mail: liujg004@ustc.edu.cn

The Serial Correlation Tests

In this paper, we do the serial correlation tests to identify the valid of estimated results. Firstly, the autocorrelation coefficient, partial autocorrelation coefficient and Q statistic of the residual series are calculated. If the corresponding p-value of the test is less than 0.05, the null of no serial correlation is rejected and, therefore, it can be concluded that there might be serial correlation in the returns [1, 2]. If the residual series of the regression equation has serial correlation, it is necessary to modify the autocorrelation of the residuals in a correct way. There is no serial correlation among all the modified regression equations, the estimated results of which are valid. The results are summarized as follows (see S1 Fig., S2 Fig., S3 Fig. and S4 Fig.)

Fig S1. Serial correlation tests between the D_BI$_{i,t}$ and Return$_{i,t}$

Fig S2. Serial correlation tests between the D_IVAS$_{i,t}$ and Return$_{i,t}$

Fig S3. Serial correlation tests between the D_BI$_{i,t}$ and Turnover$_{i,t}$

Fig S4. Serial correlation tests between the D_IVAS$_{i,t}$ and Turnover$_{i,t}$

References

1. Humala A, Rodriguez G. Some stylized facts of return in the foreign exchange and stock markets in Peru. Studies in Economics and Finance. 2013; 30(2): 139-158. doi:10.1108/10867371311325444

2. Epaphra, M. Modeling exchange rate volatility: application of the GARCH and EGARCH models. Journal of Mathematical Finance. 2017; 7(01): 121. doi:10.4236/jmf.2017.71007
