# Supplementary material for: Quantifying the effect of investors’ attention on stock market
Source: PLoS One. 2017 May 23;12(5):e0176836. doi: 10.1371/journal.pone.0176836 (PMC5441604; doi:10.1371/journal.pone.0176836)
Supplement: S1 Table — (PDF) [file pone.0176836.s003.pdf]

SUPPORTING INFORMATION S1 TABLE

Zhen-Hua Yang[1,3], Jian-Guo Liu[2,4*], Chang-Rui Yu[1*], Jing-Ti Han[2]

1 School of Information Management Engineering, Shanghai University of Finance and Economics, Shanghai 200433, PR China,
2 Data Science and Cloud Service Research Centre, Shanghai University of Finance and Economics, Shanghai 200433, PR China,
3 Business School, Huzhou University, Huzhou 313000, PR China,
4 Department of Physics, Fribourg University, CH-1700 Fribourg, Switzerland

* E-mail: liujg004@ustc.edu.cn

Table S1. Stationarity and Unit-root Tests.

Series		KPSS	p-value	ADF	p-value
$\boldsymbol{BI}_{i,t}$	CSI100	0.276	<0.01	−2.133	>0.1
$\boldsymbol{D\_BI}_{i,t}$	CSI100	0.044	>0.1	−27.839	<0.01
$\boldsymbol{IAVS}_{i,t}$	CSI100	0.378	<0.01	−3.787	<0.01
	CSI500	0.155	>0.1	−9.468	<0.01
	CSI-ALL	0.242	>0.1	−2.611	0.091
$\boldsymbol{D\_IAVS}_{i,t}$	CSI100	0.036	>0.1	−13.208	<0.01
	CSI500	0.022	>0.1	−16.025	<0.01
	CSI-ALL	0.020	>0.1	−13.462	<0.01
$\boldsymbol{Return}_{i,t}$	CSI100	0.094	0.056	−17.416	<0.01
	CSI500	0.073	>0.1	−18.523	<0.01
	CSI-ALL	0.142	>0.1	−16.342	<0.01
$\boldsymbol{D\_Return}_{i,t}$	CSI100	0.078	>0.1	−15.581	<0.01
	CSI500	0.041	>0.1	−15.469	<0.01
	CSI-ALL	0.021	>0.1	−15.145	<0.01
$\boldsymbol{Volume}_{i,t}$	CSI100	0.376	<0.01	−2.424	>0.1
	CSI500	0.318	<0.01	−2.923	0.043
	CSI-ALL	0.360	<0.01	−2.592	0.095
$\boldsymbol{D\_Volume}_{i,t}$	CSI100	0.052	>0.1	−17.113	<0.01
	CSI500	0.089	>0.1	−21.154	<0.01
	CSI-ALL	0.054	>0.1	−20.024	<0.01
$\boldsymbol{Turnover}_{i,t}$	CSI100	0.377	<0.01	−2.485	>0.1
	CSI500	0.366	<0.01	−3.138	0.025
	CSI-ALL	0.375	<0.01	−2.637	0.086
$\boldsymbol{D\_Turnover}_{i,t}$	CSI100	0.058	>0.1	−16.745	<0.01
	CSI500	0.079	>0.1	−20.923	<0.01
	CSI-ALL	0.052	>0.1	−19.958	<0.01
