# Supplementary figures and images for: Quantifying the effect of investors’ attention on stock market
Source: PLoS One. 2017 May 23;12(5):e0176836. doi: 10.1371/journal.pone.0176836 (PMC5441604; doi:10.1371/journal.pone.0176836)

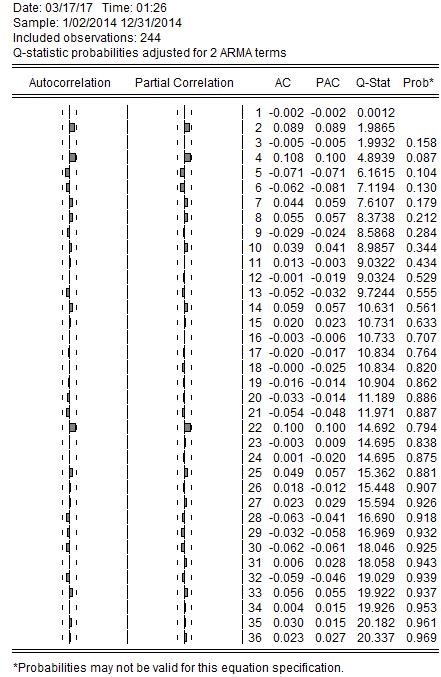

Supplement: S1 Fig — (JPG) [file pone.0176836.s004.jpg]

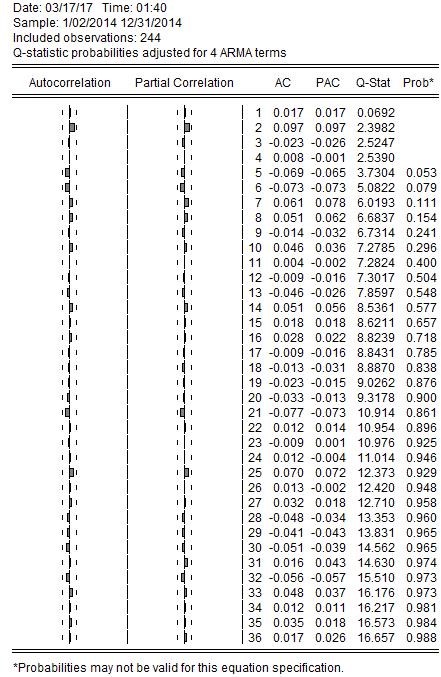

Supplement: S2 Fig — (JPG) [file pone.0176836.s005.jpg]

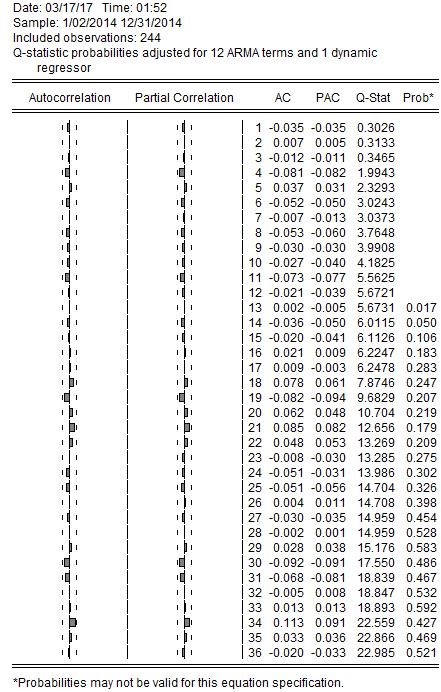

Supplement: S3 Fig — (JPG) [file pone.0176836.s006.jpg]

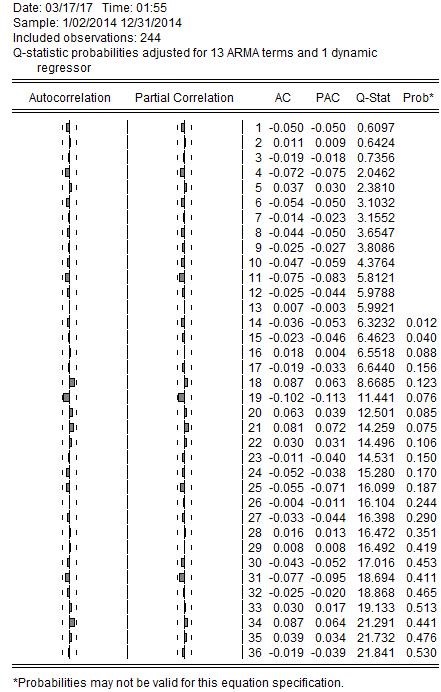

Supplement: S4 Fig — (JPG) [file pone.0176836.s007.jpg]
